# Case Report: KLHL11 encephalitis in a female patient with dual primary malignancies of breast and lung cancer: response to FcRn inhibitor therapy

**DOI:** 10.3389/fimmu.2025.1613070

**Published:** 2025-11-06

**Authors:** Yapeng Li, Haoran Xing, Zhentang Cao, Pan Cui, Lu Zhao

**Affiliations:** 1 Department of Neurology, The First Affiliated Hospital of Zhengzhou University, Zhengzhou, China; 2 National Health Commission (NHC) Key Laboratory of Prevention and Treatment of Cerebrovascular Diseases, The First Affiliated Hospital of Zhengzhou University, Zhengzhou, China; 3 Department of Oncology, The First Affiliated Hospital of Zhengzhou University, Zhengzhou, China

**Keywords:** KLHL11 antibody, autoimmune encephalitis, paraneoplastic neurological syndromes, neonatal Fc receptor inhibitor, efgartigimod

## Abstract

Kelch-like protein 11 (KLHL11) encephalitis is a rare clinical condition characterized by autoimmune-mediated encephalitis associated with the presence of KLHL11 antibodies, which commonly presents with ataxia, diplopia, vertigo, hearing loss, and seizures. While the link between this high-risk paraneoplastic autoantibody and male testicular germ cell tumors is well-established, female cases are rare, and no case of ductal breast carcinoma or dual primary malignancies have been reported. The disease is generally resistant to conventional therapies. In this study, we reported a case of KLHL11 encephalitis in a female patient presenting with fever, seizures, and ataxia, alongside dual primary malignancies: ductal breast carcinoma and pulmonary adenocarcinoma. Following immunotherapy with a neonatal Fc receptor (FcRn) inhibitor (efgartigimod) and resection of tumors, the patient achieved complete symptomatic remission with no recurrence. Current studies showed that KLHL11 encephalitis contributed to pathogenesis through cytotoxic T-cell-mediated neuronal injury and loss. However, in this case, rapid clinical improvement was observed after FcRn inhibitor therapy. This is the first report of FcRn inhibitor in the treatment of KLHL11 encephalitis. This case demonstrated a rare association of KLHL11 encephalitis with breast and lung cancer, and expanded on the clinical manifestations, associated tumor types, and treatment options of KLHL11 encephalitis in women.

## Introduction

Autoimmune Kelch-like protein 11 (KLHL11) encephalitis was first reported in 2019 (1). The clinical manifestations include cerebellar syndrome and/or brainstem involvement, presenting with symptoms such as vertigo, hearing loss, diplopia, dysarthria, and ataxia. This novel antibody can be detected in serum and cerebrospinal fluid (CSF) using tissue-based and cell-based assays. Due to intracellular localization of KLHL11 protein, the pathogenic mechanism of KLHL11 encephalitis is considered to be T-cell-mediated (1). The syndrome shows a predilection for adult males, a strong association with testicular germ cell tumors, and is generally refractory to immunotherapy and tumor treatments (2). KLHL11 encephalitis exhibits a strong association with malignancies and is classified as a paraneoplastic syndrome (PNS). To date, few female cases have been reported in the literature, and no instances of ductal breast carcinoma or dual primary malignancies have been documented in patients with KLHL11 encephalitis.

In this case, we reported a female patient presenting with fever, seizures and ataxia. The KLHL11 IgG antibody was positive in serum and CSF. The patient achieved complete symptomatic remission following four weekly infusions of efgartigimod, a neonatal Fc receptor inhibitor (FcRn). Intravenous efgartigimod received its first approval in 2021 for the treatment of generalized myasthenia gravis in adults with positive anti-AChR antibody (3). Efgartigimod effectively reduces the levels of pathogenic IgG, and alleviates T cell activation and the production of systemic pro-inflammatory cytokines (4, 5). The chest computed tomography (CT) revealed a small nodule in the right lung. The whole-body fluorine-18 fluorodeoxyglucose (^18^F-FDG) positron emission tomography/computed tomography (PET/CT) revealed a hypermetabolic focus in the left breast upper outer quadrant. The patient underwent a partial mastectomy, and postoperative pathology revealed moderate-grade ductal carcinoma *in situ*. At the 8-month follow-up after disease onset, the patient remained clinically asymptomatic, with serum KLHL11 IgG antibodies still detectable. Additionally, a chest CT scan demonstrated an increase in the size of the right lung nodule with an indistinct border. Thoracoscopic partial lung resection was performed, and the postoperative pathology showed pulmonary adenocarcinoma *in situ*. The patient had complete symptomatic remission and no recurrence following immunotherapy and tumor resection so far.

## Case description

A 34-year-old female patient was admitted to our hospital on April 18, 2024, due to subfebrile temperature and seizures. Two days prior to admission, she had developed a low-grade fever, with a maximum temperature of 37.6°C, in the absence of respiratory infection symptoms. There were two episodes of epileptic seizures within 10 hours, characterized by loss of consciousness and involuntary twitching of limbs, with each episode lasting 2–3 minutes. The patient reported no history of toxin exposure, smoking, or alcohol consumption, and had no significant past medical or surgical history. Family history was unremarkable, with no hereditary or similar conditions reported among first-degree relatives. Physical examination revealed nuchal rigidity and positive right Babinski sign.

**Figure 1:**
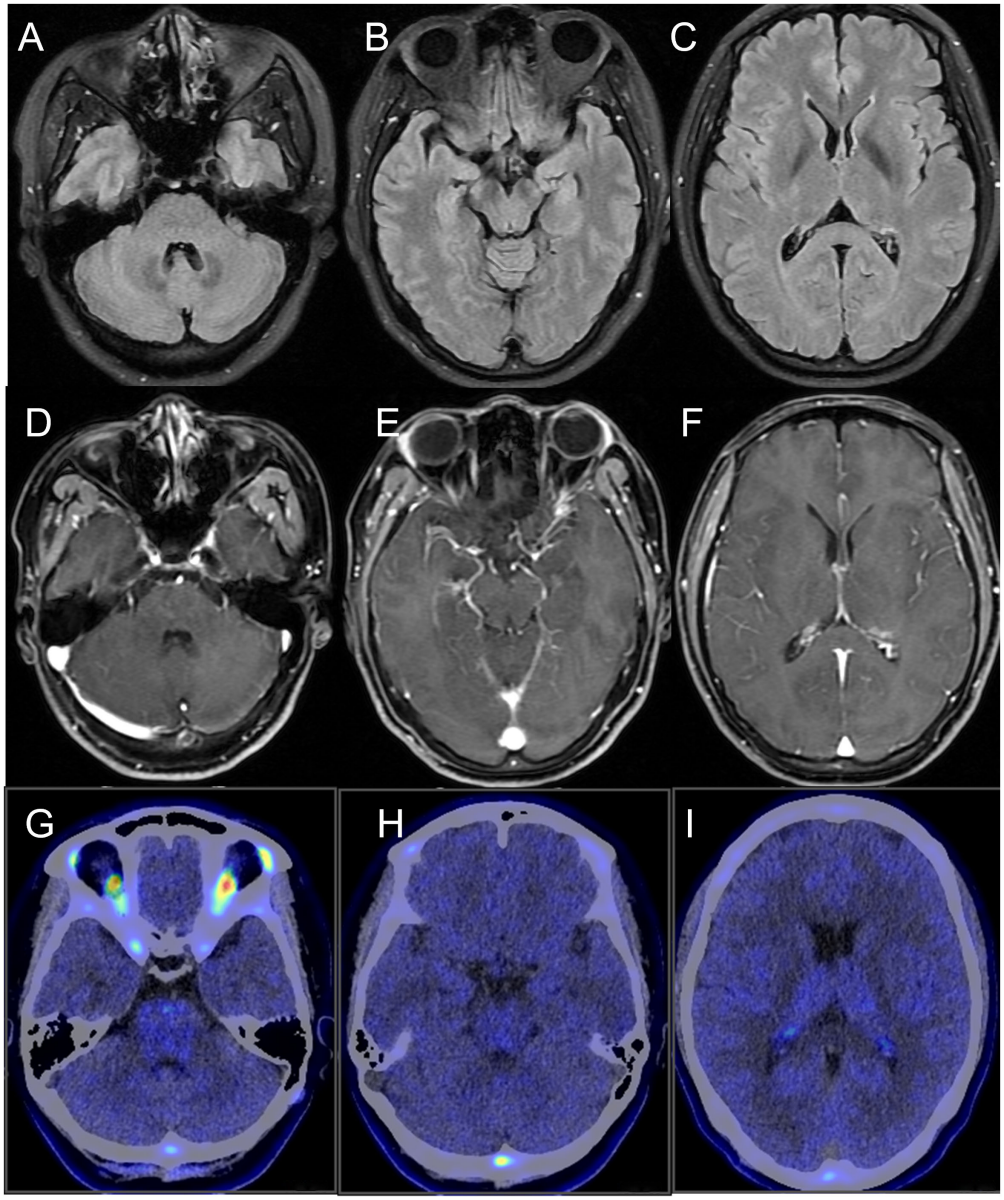
Neuroimaging of the patient. **(A-C)** brain MRI fluid-attenuated inversion recovery sequence; **(D-F)** brain MRI T1-weighted contrast-enhanced sequence; **(G-I)** brain 18F-DPA714 PET/CT. No abnormalities were detected.

**Figure 2:**
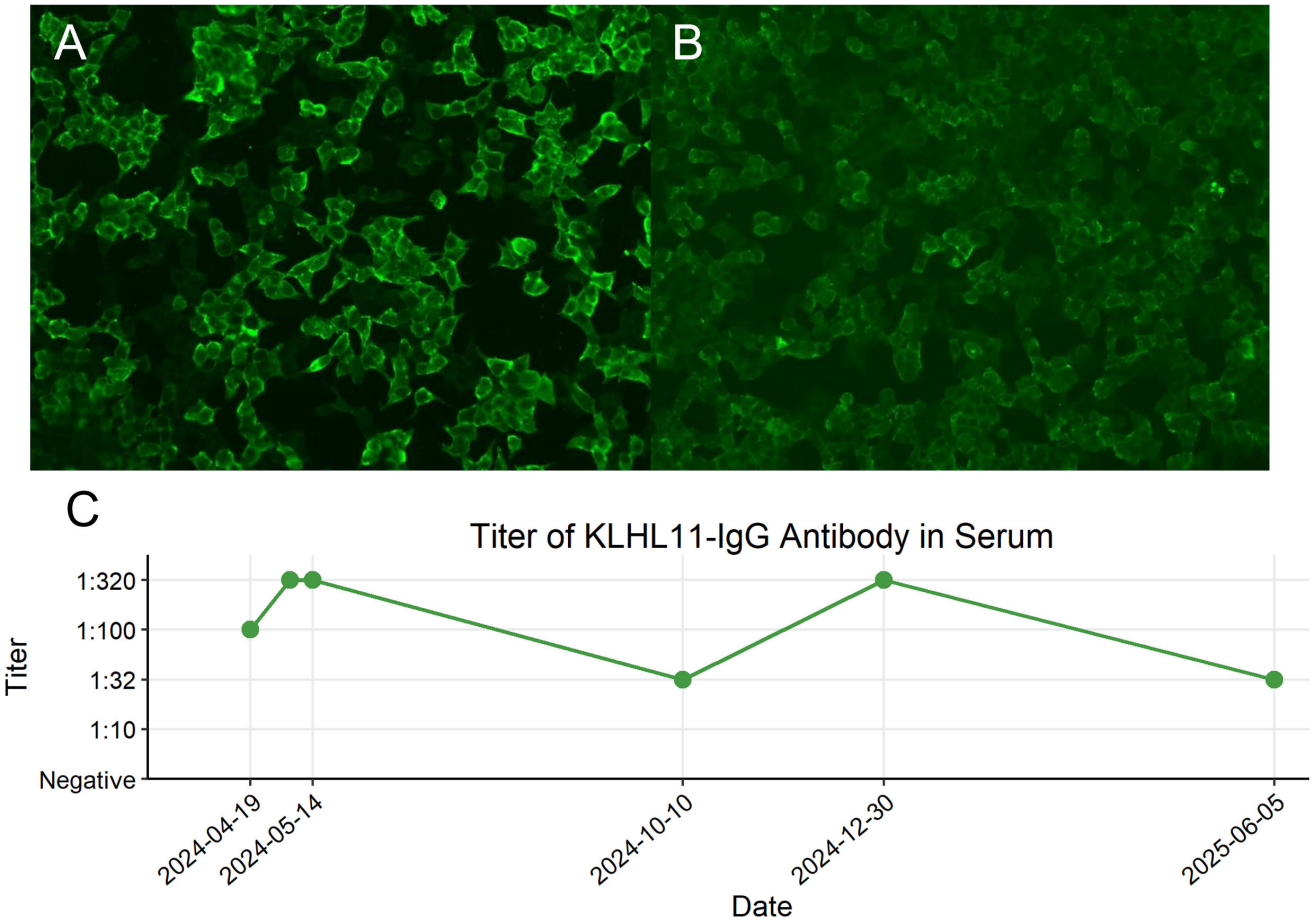
Cell-based assay fluorescence images demonstrating KLHL11 IgG positivity in serum and cerebrospinal fluid (CSF) and longitudinal monitoring of serum KLHL11-IgG titers. **(A)** Fluorescent antibody staining showing KLHL11 antibody expression in the first serum sample (titer 1:100). **(B)** Fluorescent antibody staining showing KLHL11 antibody expression in the first CSF sample (titer 1:3.2). **(C)** Longitudinal changes in serum KLHL11-IgG titers during the clinical course.

No abnormalities were detected on brain MRI (**Figures 1A-F**). The routine and 24-hour long-term electroencephalogram were normal during the interictal period. Analysis of CSF showed normal intracranial pressure (120 mmH_2_O), normal leukocytes (2×10^6^), low protein (138.1mg/L), normal glucose (3.27mmol/L) and normal chloride (125.6mmol/L). CSF metagenomic next-generation sequencing was negative. Serum and CSF tissue-based immunofluorescence assays were positive within neuronal cells of cerebellum and hippocampus. KLHL11 IgG antibody by cell-based assay was positive in serum (1:100) and CSF (1:3.2) (**Figure 2**).

The serum tumor markers (carcinoembryonic antigen, β-human chorionic gonadotropin, alpha-fetoprotein, tumor-associated antigen 125, tumor-associated antigen 153, tumor-associated antigen 199, tumor-associated antigen 72-4, CYFRA 21-1, neuron-specific enolase, and ferritin) and serum paraneoplastic markers (Hu, Yo, Ri, CV2, Ma2, and Amphiphysin) were negative. The serum inflammatory indicators and autoimmune antibodies were negative. The breast ultrasound revealed a hypoechoic nodule in the left breast, and the mammogram identified a calcified lesion at the same location. Both breast imaging findings were assigned a BI-RADS Category 3 classification, indicating a probably benign lesion. The chest CT revealed a small nodule (<5mm) in the right lung, with a low likelihood of malignancy. The whole-body ^18^F-FDG-PET/CT identified a hypermetabolic focus in the left breast upper outer quadrant (SUV_max_: 5.6). The brain ^18^F-DPA714-PET/CT showed no significant increase of translocator protein expression (**Figures 1G-I**).

**Figure 3:**
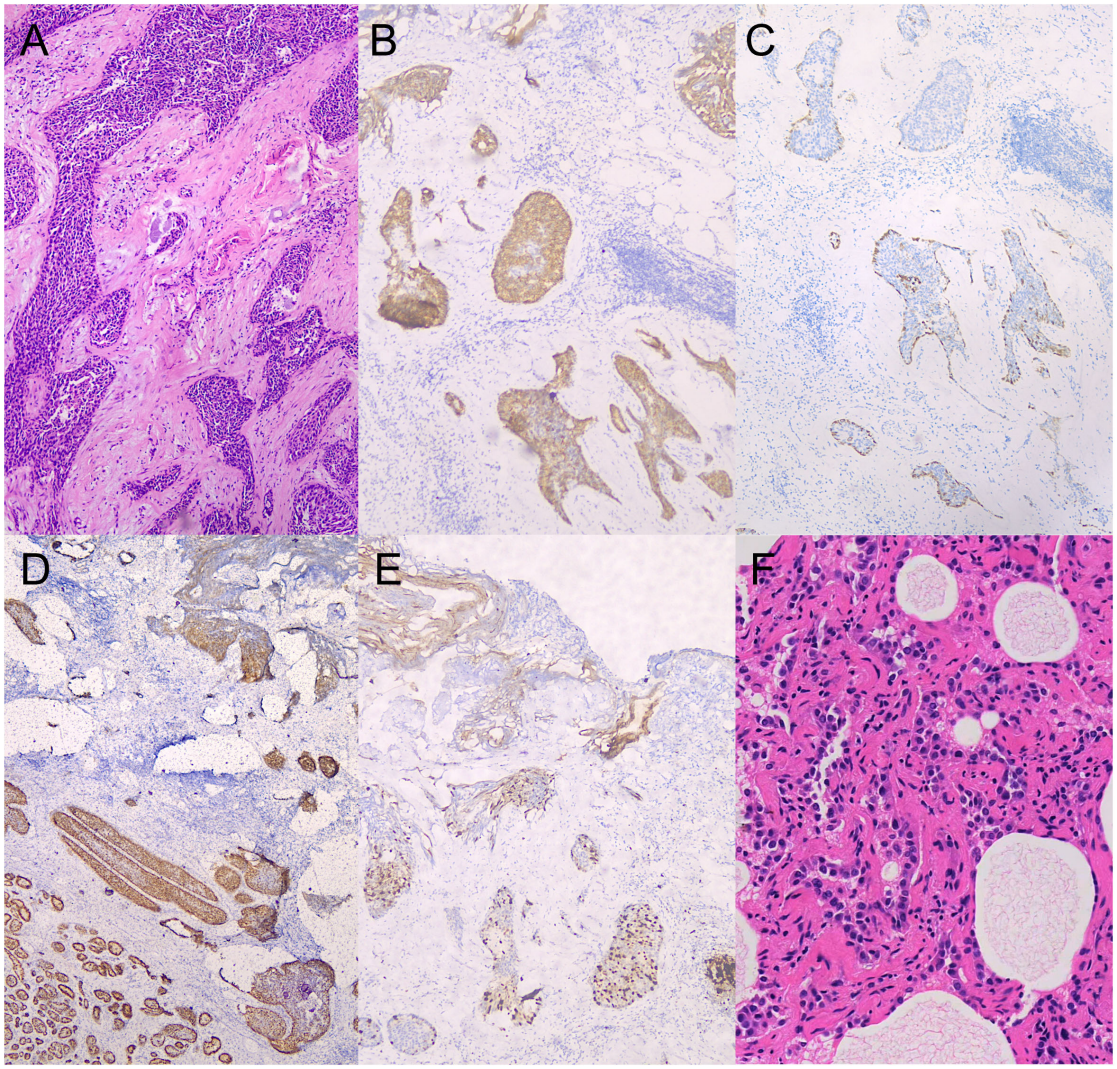
Pathological findings of breast and lung cancer (40× magnification). **(A)** ductal breast carcinoma *in situ* (hematoxylin-eosin stain). **(B)** human epidermal growth factor receptor 2 (HER2/neu) immunohistochemical staining of breast cancer. **(C)** CK-14 immunohistochemical staining of breast cancer. **(D)** Estrogen receptor (ER) immunohistochemical staining of breast cancer. **(E)** Ki-67 proliferation index immunohistochemical staining of breast cancer. **(F)** lung adenocarcinoma *in situ* (hematoxylin-eosin stain).

The patient was diagnosed with KLHL11 encephalitis, and received levetiracetam on April 19, 2024 and efgartigimod on April 21. Despite the recommendation, the patient declined glucocorticoid therapy and plasmapheresis due to concerns about potential side effects. However, after discharge, she developed fever on April 27 with the highest temperature reaching 38.1°C. Additionally, she experienced dizziness and gait instability, scoring 2 points on the scale for the assessment and rating of ataxia (SARA). The infectious fever was excluded and she continued receiving weekly efgartigimod. She received a total of four weekly infusions of efgartigimod. There was no recurrence of fever, and her dizziness and ataxia were relieved. The SARA score was 0 after the fourth infusion of efgartigimod. Ultrasound-guided fine needle aspiration biopsy was performed, and the pathological result showed focal atypical ductal hyperplasia. Given the suspicion of malignancy, the patient underwent a partial mastectomy on May 29. The postoperative pathological diagnosis revealed moderate-grade ductal carcinoma *in situ* in the left breast. Immunohistochemical analysis showed estrogen receptor (ER) positivity in 80% of cells (+++), progesterone receptor (PR) positivity in 10% of cells (+), human epidermal growth factor receptor 2 (HER2/neu, CerbB-2) overexpression (+++), and Ki-67 proliferation index of 15% (+) (**Figure 3**). Subsequently, she received intravenous immunoglobulin at a dose of 0.4 g/kg/day for 2 days for prevention of autoimmune encephalitis relapse, followed by a total of 20 fractions of radiotherapy to the left breast.

**Figure 4:**
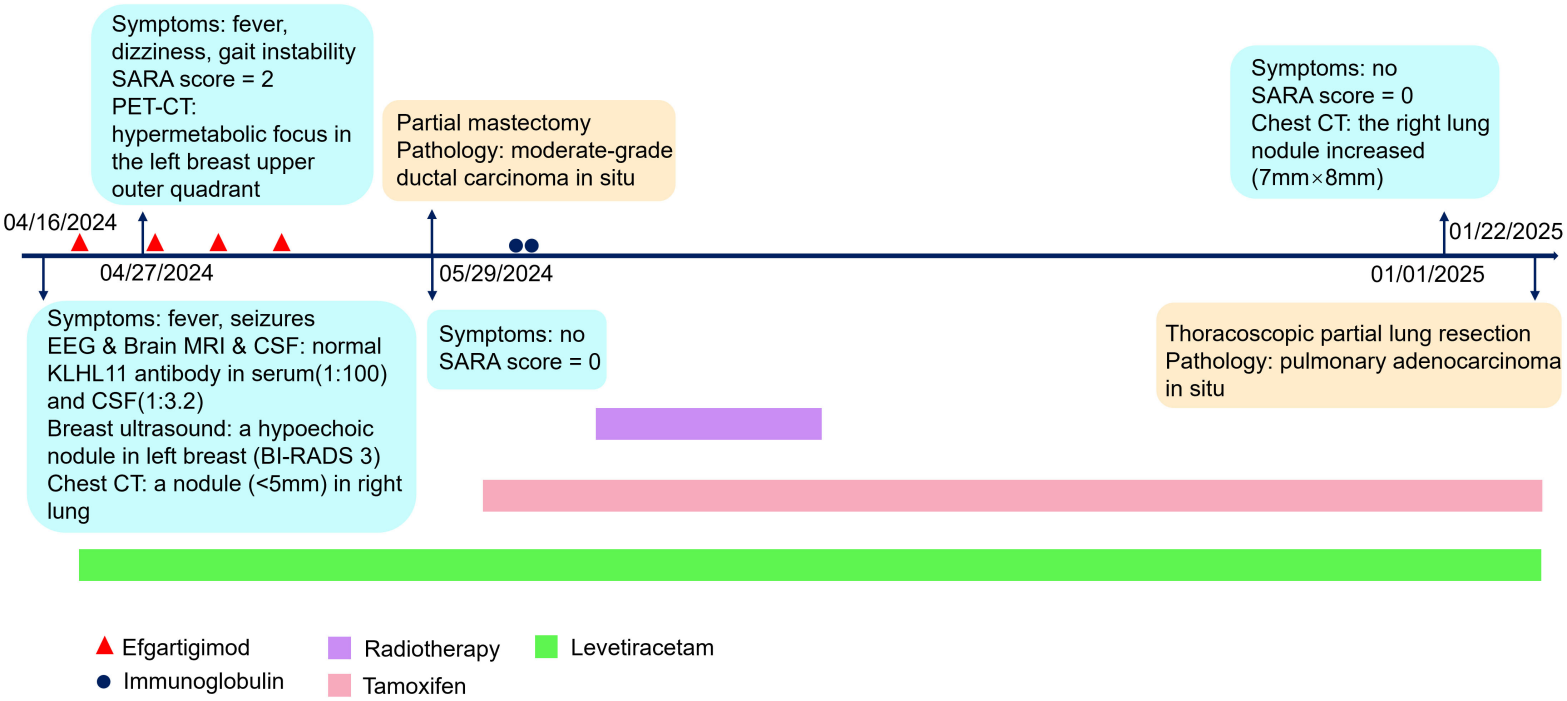
Timeline of disease and treatment.

The patient was asymptomatic, and took levetiracetam and tamoxifen regularly. There was no recurrence of fever, epilepsy or ataxia. No adverse events occurred. Serum KLHL11 IgG antibodies were re-evaluated in the same lab, and remained detectable. Longitudinal monitoring of serum KLHL11-IgG titers was presented in **Figure 2C**. The follow-up chest CT scan conducted on January 1, 2025, revealed an increase in the size of the right lung nodule (7mm×8mm), with an indistinct border, suggesting a high likelihood of malignancy. Thoracoscopic partial lung resection was performed on January 22. The postoperative pathology showed pulmonary adenocarcinoma *in situ*. The clinical manifestations, diagnosis and treatment of the patient was presented in detail in **Figure 4**.

## Discussion

This case was a young female patient with KLHL11 encephalitis presenting with fever, seizures and ataxia. She was pathologically diagnosed with dual primary malignancies consisting of ductal breast carcinoma and pulmonary adenocarcinoma. Her clinical symptoms were quickly relieved following immunotherapy utilizing FcRn inhibitor, and there was no recurrence after tumor resection.

KLHL11 encephalitis was initially identified in a male patient with seminoma (1). Epidemiological data indicate a male predominance, with a male-to-female ratio of approximately 5.6:1 (6). As a paraneoplastic neurological syndrome, the condition is frequently associated with malignancies, with approximately 71% of affected patients having tumors (6). Testicular germ cell tumors are the most common, followed by teratoma in testicle or ovary (7). Other associated tumors include small cell lung cancer, lung adenocarcinoma, ovarian carcinoma, chronic lymphocytic leukemia, Müllerian tumor, and esophageal cancer (7–9). In this case, the patient had dual primary malignancies, and we considered that the radiotherapy of ductal breast carcinoma was unrelated to the subsequent pathologically confirmed lung adenocarcinoma, as the radiotherapy was localized, the breast and lung tumors were on contralateral sides, and the lung nodule was already evident on the initial chest CT scan. Routine screening for tumors should be conducted in patients with KLHL11 encephalitis, and whole-body ^18^F-FDG PET/CT imaging can be helpful in detecting potential tumors. However, if no tumor is identified initially, close monitoring is necessary, as PET/CT may fail to detect small lesions. In this case, the dual malignancies of the patient were in the very early stage, and the initial whole-body PET/CT did not reveal the lung tumor. Notably, to date, no cases of KLHL11 encephalitis co-occurring with breast cancer have been documented in the literature, and the present case represents the first documented instance of KLHL11 encephalitis occurring concomitantly with both ductal breast carcinoma and primary lung adenocarcinoma. However, a definitive causal relationship between the antibody and either malignancy cannot be established due to the absence of direct immunohistochemical evidence of KLHL11 antigen expression in tumors. Mammectomy was performed at an external institution, and further immunohistochemical studies are planned pending coordinated access to archival tissue blocks from both treating institutions.

The most common clinical phenotype of KLHL11 encephalitis is rhombencephalitis (brainstem and/or cerebellar involvement), followed by cochleovestibulopathy and limbic encephalitis (7). The clinical presentations include ataxia, diplopia, vertigo, hearing loss, tinnitus, dysarthria, and seizures. Notably, hearing loss or tinnitus can precede other neurologic deficits by several months (2). The clinical spectrum was more heterogeneous than reported previously. Atypical neurologic presentations encompass neuropsychiatric dysfunction, hypersomnia, myeloneuropathy, trigeminal neuralgia, opsoclonusmyoclonus, parkinsonism, cervical amyotrophy and weight loss (1, 2, 10).

MATCH score (male, ataxia, testicular cancer and hearing alterations) was designed to facilitate the identification of patients that should be tested for KLHL11 antibodies with a cut-off ≥ 4 points (10). A previous study showed the sensitivity of MATCH score was 78%, and the specificity was 99% (11). Our patient had a MATCH score of 2 (ataxia=1 and other cancer=1), indicating the score was not sensitive for identifying KLHL11 encephalitis patients, especially female patients. Therefore, when screening for KLHL11 encephalitis, the MATCH score should be interpreted with caution. We suggest that all patients with rhombencephalitis of unknown infectious or metabolic etiology should be screened for KLHL11 antibody, as a positive result would enable timely initiation of immunotherapy, potentially preventing irreversible neurological deficits. Additionally, more comprehensive tumor screening and monitoring would be promoted. With increasing recognition of the disease and a growing number of reported cases of KLHL11 encephalitis, more sensitive screening tools are likely to be developed.

To optimize the sensitivity and specificity for KLHL11 encephalitis diagnosis, a combination of the cell-based assay (CBA) and tissue-based assay (TBA) is recommended. KLHL11 antibody detected by CBA alone might lead to false positive results (2). Some KLHL11 encephalitis patients coexisted with other paraneoplastic antibodies, including anti-leucine zipper 4, anti-Ma2 and anti-Hu. The titer of KLHL11 antibody by TBA in serum was higher than that in CSF (1, 2). According to the PNS-Care panel’s expert advice, paired serum and CSF samples tests were first recommended, and serum was preferred if paired samples were unavailable (12). However, the changes in antibody titer following immunotherapy and/or oncotherapy and their relationship with disease severity remained unknown. Two case reports demonstrated that the patients’ symptoms were alleviated following treatment, and the titer of KLHL11 antibodies decreased or became negative during the follow-up period (13, 14). In our present case, the most recent titer (measured at Month 13 post-diagnosis) showed a significant decline. Notably, no reduction in titer was observed despite clinical improvement after four weekly infusions of efgartigimod administration. This discrepancy may be attributed to persistent antibody production from tumor-driven immune activation in this paraneoplastic syndrome. And this phenomenon suggests that the therapeutic benefit of efgartigimod might involve immunomodulatory effects beyond IgG reduction.

In this case, the neurological deficits were relieved rapidly following administration of FcRn inhibitor, and the condition remained stable after tumor resection. This is the first report of FcRn inhibitor in the treatment of KLHL11 encephalitis. To date, there is no standard treatment for the KLHL11 encephalitis, and current management strategies are adapted from other PNS, including immunotherapy and underlying tumor management (15). Traditional first-line immunotherapies include intravenous methylprednisolone, intravenous immunoglobulin and plasma exchange, and second-line immunotherapies consist of cyclophosphamide, rituximab, mycophenolate mofetil, azathioprine, tacrolimus, and natalizumab (7). Most patients with KLHL-11-PNS were refractory to treatment. Progressive neurological function decline occurred in 48.1% of patients treated with immunotherapy and/or oncotherapy, stabilization in 23.1%, and improvement in 28.8% (7). Our patient declined glucocorticoids and plasmapheresis. Based on emerging reports with efgartigimod in autoimmune encephalitis (16, 17), the patient consented to efgartigimod after being informed of its potential therapeutic benefit, under our institution’s off-label use protocol.

The intracellular localization of KLHL11 protein probably precludes a direct role of KLHL11 IgG in neuronal cell death and injury. KLHL11 encephalitis primarily contributes to pathogenesis through T-cell-mediated inflammatory responses and specific T-cell reactions (1, 7, 10). KLHL11 IgG may trigger immune attacks against neurons and tumor cells by activating antigen-presenting cells (such as dendritic cells) and T-cell responses (1). Infiltrated T-cell-mediated immune damage on CNS led to a two-stage disease course, consisting of severe inflammation at the active/early stage (reversible) and cumulative neuronal loss at the progressive/advanced stage (irreversible) (7). Efgartigimod is an FcRn inhibitor that treats autoimmune diseases by reducing circulating IgG antibody levels, thereby reducing the levels of pathogenic IgG autoantibodies, as in generalized myasthenia gravis and IgG4-related disease (3, 18). Furthermore, efgartigimod suppresses FcRn-regulated antigen-IgG immune complex (IC) presentation in antigen-presenting cells, e.g., dendrite cells and macrophages, thus damping MHC II-mediated CD4^+^ T cell activation and MHC I-mediated CD8^+^ T cell cytotoxicity. Meanwhile, IgG-IC-mediated complement activation is attenuated. On the other hand, efgartigimod impedes the release of pro-inflammatory cytokines (e.g., IL-6, TNF-α, and IFN-γ), which is triggered by IgG binding to Fcγ receptors (FcγRs) on macrophages and dendritic cells. Collectively, efgartigimod effectively restrains massive inflammatory cascades by targeting both adaptive and innate immune system, as well as both humoral and cellular immunity (4, 5).

We hypothesized that the favorable treatment outcome in our patient might be attributed to the fact that both the PNS-encephalitis and the tumor were in the very early stages, and the FcRn inhibitor effectively reduced the pathogenic KLHL11 IgG level and alleviated autoreactive T cell activation and the production of systemic pro-inflammatory cytokines. Therefore, the timely initiation of combined immunological and oncological therapy may help mitigate irreversible neuronal damage and prevent severe neurological disability.

## Conclusion

Although KLHL11 encephalitis is rare in women, it should be considered in all patients with rhombencephalitis in absence of infectious or metabolic etiologies. This case demonstrates an association between KLHL11 encephalitis and dual malignancies (ductal breast carcinoma and pulmonary adenocarcinoma), expanding the known tumor spectrum in female patients. It also suggests the potential efficacy of FcRn inhibitors in treating the syndrome and emphasizes the significance of early recognition and intervention in KLHL11 encephalitis. Further studies are needed to validate the association between KLHL11 antibodies and dual malignancies and to evaluate FcRn inhibitor efficacy in larger cohorts.

## Data Availability

The original contributions presented in the study are included in the article/supplementary material. Further inquiries can be directed to the corresponding author.
